# Long‐term cost‐effectiveness of a more accurate diagnostic work‐up for dementia

**DOI:** 10.1002/dad2.70210

**Published:** 2025-11-04

**Authors:** Pieter J. van der Veere, Hana M. Broulikova, Jeroen Hoogland, Ingrid S. van Maurik, Elsmarieke van de Giessen, Argonde C. van Harten, Judith E. Bosmans, Johannes Berkhof, Wiesje M. van der Flier

**Affiliations:** ^1^ Alzheimer Center and Department of Neurology Amsterdam Neuroscience, Amsterdam UMC Amsterdam the Netherlands; ^2^ Department of Epidemiology and Biostatistics Amsterdam Neuroscience, Amsterdam UMC Amsterdam the Netherlands; ^3^ Amsterdam Neuroscience, Neurodegeneration Amsterdam the Netherlands; ^4^ National Institute of Mental Health Klecany Czech Republic; ^5^ Department of Health Sciences, Faculty of Science Vrije Universiteit Amsterdam Amsterdam the Netherlands; ^6^ Northwest Academy, Northwest Clinics Alkmaar Alkmaar the Netherland; ^7^ Department of Radiology & Nuclear Medicine Vrije Universiteit Amsterdam, Amsterdam University Medical Center Amsterdam the Netherlands

**Keywords:** Alzheimer's disease, cost‐effectiveness analysis, diagnostic utility, institutionalization, positron emission tomography

## Abstract

**INTRODUCTION:**

To address uncertainty about long‐term clinical and economic impacts of an accurate dementia diagnosis, we evaluated the cost‐effectiveness of adding amyloid positron emission tomography (PET) to memory clinic workup over 5 years.

**METHODS:**

Inverse probability weighting was used to balance covariates between PET (*n* = 440) and no‐PET (*n* = 460) participants from the Amsterdam Dementia Cohort. Time in community following diagnosis, time alive, and costs were combined in cost‐effectiveness analyses.

**RESULTS:**

PET participants lived longer in community (0.26 years, 95% confidence interval [CI]: 0.05 to 0.45) and overall (0.15, CI: 0.02 to 0.27), but did not have statistically different health insurance (€703, CI: −3974 to 5045) or total costs including institutionalization (−€8258, CI: −20,622 to 3377). The probability that PET was cost‐effective for extending time in community was 76% at a €2530 willingness‐to‐pay threshold. The probability that PET yielded cost savings and was more effective for extending time alive was 90%.

**DISCUSSION:**

Findings in this observational cohort suggest that using amyloid PET in memory clinics may be cost‐effective.

**Highlights:**

- Participants with an amyloid PET in a memory clinic work‐up were compared to those without.
- The amyloid PET group spent more time in community and alive over 5 years of follow‐up.
- Amyloid PET had a 76% chance to cost‐effectively extend time in community in uncertainty analysis.

## 1 INTRODUCTION

Worldwide, about 55 million people are living with dementia, two‐thirds of whom have Alzheimer's disease (AD). Dementia poses an enormous emotional as well as economic burden for people living with the disease, their families, and society. In 2019, the global annual costs reached US$1313.4 billion, corresponding to $23,796 per person living with dementia.^1^ To date, no available treatments cure or stop the disease, although the first disease‐modifying treatments for AD with modest effect have gained market access.^2^ In addition, preventive strategies and tailored disease management programs may mitigate and delay the impact of the disease and decrease its burden.^3^, ^4^, ^5^, ^6^, ^7^

An accurate diagnosis is an important prerequisite to initiate effective management of AD,^8^, ^9^, ^10^, ^11^ which is indicated to improve patient care.^7^ Adding diagnostic information from biomarkers in cerebrospinal fluid (CSF) or amyloid PET enable a more accurate etiological diagnosis of AD and increase clinician confidence in the diagnosis.^12^, ^13^, ^14^ This is also supported by randomized controlled trial evidence, as the AMYPAD trial showed that amyloid PET increased diagnostic certainty after 12 weeks.^15^ Thus, improving the diagnostic process of AD through the addition of amyloid PET may improve patient outcomes.

As some benefits of an improved diagnostic process may manifest over longer timeframes, it is imperative to study longer‐term clinical and economic consequences of adding amyloid PET to the diagnostic work‐up. In our recent cohort study incorporating amyloid PET into the diagnostic work‐up, we found lower rates of institutionalization and mortality and a decrease in annual costs over a 4‐year follow‐up period in the group with an amyloid PET compared to the group without an amyloid PET.^16^ The Imaging Dementia‐Evidence for Amyloid Scanning (IDEAS) registry‐based study of Medicare patients with 1‐year follow‐up observed a decrease in hospitalizations among dementia patients with an amyloid PET compared to matched controls but no significant difference among patients with mild cognitive impairment (MCI). Follow‐up Medicare costs for both MCI and dementia patients were lower compared to the matched controls. However, the total cost difference over the year of follow‐up was smaller than the cost of PET scans, leading to higher overall costs for patients who received PET.^17^ Additionally, while three studies relying on decision analytic models to investigate long‐term cost‐effectiveness concluded that PET had the potential to improve patient outcomes and reduce costs,^18^, ^19^, ^20^ the most recent modeling study in this field found the opposite.^21^ Differences in the results were partially attributed to different assumptions regarding the long‐term effects of an amyloid PET due to limited long‐term clinical and cost data.

Together with the relatively high costs of amyloid PET,^17^ the uncertainty regarding the long‐term utility of adding amyloid PET to the diagnostic work‐up drives the discussion regarding its wider implementation. To address this gap, we investigated the cost‐effectiveness of improving diagnostic accuracy by adding amyloid PET to the memory clinic diagnostic workup over a 5‐year period.

## 2 METHODS

### 2.1 Study population and data

We used data from a previously established and described sample consisting of Amsterdam Dementia Cohort participants who took part in the ABIDE‐PET project and for whom we acquired additional follow‐up data from Statistics Netherlands.^16^, ^22^ In short, between October 27, 2014, and December 31, 2016, all 1076 patients visiting the Alzheimer Center Amsterdam memory clinic with cognitive complaints were offered the option to undergo an amyloid PET as part of their baseline visit. We obtained written informed consent from all participants. Six hundred participants who did not opt for additional PET scan (no‐PET group) underwent the standardized work‐up, which includes demographic information, medical history, neurological, physical as well as neuropsychological tests, magnetic resonance imaging (MRI), and a lumbar puncture.^23^ For the 476 participants who participated in the amyloid PET study (PET group), the PET scan was performed in addition to the standardized work‐up on the same day. The 18F‐florbetaben (FBB; Neuraceq) scans were conducted 90 to 110 min after injection using 3T Philips Ingenuity TF PET/MR, Philips Ingenuity TF PET/CT, or Philips Gemini TF PET/CT scanners, following approved protocols. Amyloid positivity was determined by visual assessment of the amyloid PET by an experienced nuclear medicine physician masked to clinical information.^10^ Clinical diagnoses for all participants were made during a multidisciplinary meeting, determining the clinical stage (subjective cognitive decline [SCD], MCI, dementia), and the most likely etiology based on available criteria.^24^, ^25^, ^26^, ^27^, ^28^

**RESEARCH IN CONTEXT**

1. **Systematic review**: Previous studies suggested that more accurate diagnoses at the memory clinic could improve patient outcomes through more targeted care. However, studies investigating cost and clinical outcomes after an amyloid PET scan do not consistently find it to be a cost‐effective addition to the diagnostic work‐up in a memory clinic.
2. **Interpretation**: We investigated the cost‐effectiveness of improving diagnostic accuracy by adding amyloid PET to the memory clinic diagnostic work‐up over a 5‐year period. Patients with an amyloid PET spent more time in community and more time alive compared to those without a PET. The addition of an amyloid PET is likely to be cost‐effective at lengthening the time in the community and yield cost savings at extending time alive.
3. **Future direction**: These observational results support the hypothesis that a more accurate diagnosis improves patient outcomes and should be confirmed in randomized controlled trials with sufficient follow‐up to identify an effect.

In total, 1020 participants could be probabilistically linked with the registry data managed by Statistics Netherlands (37 patients did not give consent for the linkage, and probabilistic linkage failed for 19 participants). The purpose of the linkage was to obtain information on date of institutionalization, death, and annual healthcare costs until death or December 31, 2021, whichever event occurred earlier. Due to the unavailability of the cost data for the year 2014, only participants joining the ABIDE‐PET cohort from January 1, 2015, were considered in this study.

### 2.2 Outcomes

The effect outcomes for this cost‐effectiveness analysis were time in the community (i.e., time before institutionalization or death) and time alive following the diagnosis. The cost outcomes included costs of the PET scan and healthcare costs reimbursed under the health insurance act, disaggregated into the following cost categories: primary care (includes general practitioner costs), hospital‐related care (includes in‐patient, out‐patient hospital, and geriatric rehabilitation costs), mental health care, aid products (e.g., crutches), medication, homecare, and other (see Supplement 1 for a description and Dutch labels). Costs of institutionalization were calculated by multiplying the daily costs of living in the institution in the Netherlands (€290 per day) by the number of days spent in the institution.^29^ For the PET scan, one‐time intervention costs of €2500 were added to the costs in the PET group.^17^ Costs were indexed to 2021 euros. For the outcome time in the community, healthcare costs excluding cost of institutionalization accumulated over 5 years were considered (hereafter called health insurance costs). For the outcome time alive, both health insurance and institutionalization costs were considered (hereafter called total costs).

### 2.3 Statistical analysis

**FIGURE 1:**
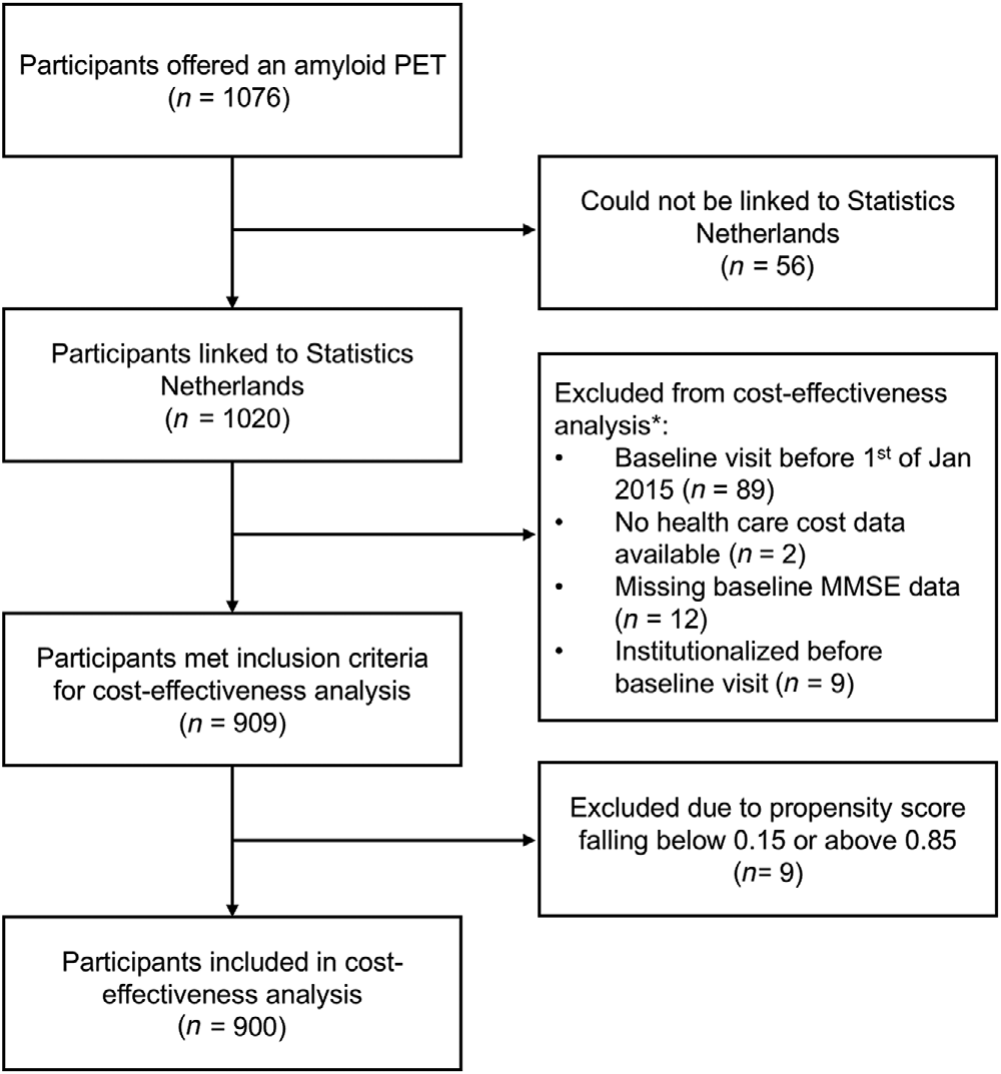
Flowchart of sample selection. * Criteria can overlap. MMSE, Mini‐Mental State Examination.

To balance the PET and no‐PET groups, inverse probability weighting was performed, calculating weight based on age, sex, syndrome diagnosis (SCD, MCI, or dementia), cognitive performance measured by Mini‐Mental State Examination (MMSE), level of education, availability of CSF biomarkers, and Charlson Comorbidity Index (a higher score indicates more comorbidities).^30^, ^31^ Overlap of propensity scores was assessed and nine propensity scores below 0.15 and above 0.85 and were excluded, after which overlap was achieved (Figure S1). See supplemental methods for further details on the propensity scores. Balance of the covariates between the groups was assessed using absolute standardized mean differences. The exposure of interest was amyloid PET scan receipt, which was controlled for potential confounding by the availability of CSF testing. Remaining imbalances in CSF amyloid positivity were checked by calculating the proportion of amyloid‐positive participants in each group after weighting. The utilized CSF assays and cut‐offs are reported elsewhere.^32^ Figure 1 presents the sample selection resulting in the study population of 440 unique PET and 460 no‐PET participants.

For the analyses, time in the community and time alive were defined as restricted mean survival time over the first 5 years of follow‐up, as for this period, no participants were administratively censored. Incremental cost‐effectiveness ratios (ICERs) were calculated by dividing the difference in accumulated costs between the PET and no‐PET groups by the difference in time in the community and time alive. Uncertainty surrounding the differences in health outcomes and costs was estimated using bootstrapping with 5000 repetitions. Subsequently, the 5000 bootstrapped cost–effect pairs were plotted on cost‐effectiveness planes to show the uncertainty surrounding the ICERs.^33^ On the cost‐effectiveness plane, the difference in effects is plotted on the horizontal axis and the difference in costs on the vertical axis resulting in four quadrants: northeast, where intervention (PET) is more expensive and more effective than control (no‐PET); southeast, where intervention is less expensive and more effective than control; southwest, where intervention is less expensive and less effective than control; and northwest, where intervention is more expensive and less effective than control. Cost–effect pairs falling in the east quadrants reflect a longer time in the community or alive in the PET compared to no‐PET group. Based on the bootstrapped cost–effect pairs, cost‐effectiveness acceptability curves were estimated to show the probability that PET was cost‐effective compared to no‐PET for a range of willingness to pay (WTP) values. No official WTP threshold is defined for prolonging time living in the community. However, in a previous study, we elicited a WTP of 3 months postponed institutionalization in addition to a more precise diagnosis of €2530 from healthy participants acting as patients with MCI.^34^ To enable direct comparison with this threshold, we scaled the cost‐effectiveness acceptability curve of time in the community to reflect WTP values for an additional 3 months in the community rather than 1 year.

To assess the robustness of the results, a sensitivity analysis was performed that presented results based on an alternative method to balance the PET and no‐PET groups, namely, propensity score matching as employed in the previous study based on these data.^16^ In the prior study, PET and no‐PET participants were matched on propensity scores calculated with the same covariates. No‐PET participants were selected with replacement, that is, a no‐PET participant could be matched to multiple PET participants.

All analyses in this study were performed using R programming software version 4.2.3,^35^ using the “survival” package.^36^

## 3 RESULTS

Of the 900 participants, 392 (44%) were female, with an average age (SD) of 63 (8) years. At baseline, 364 (40%) had SCD, 135 (15%) had MCI, and 401 (45%) dementia (Table 1). After application of inverse probability weighting, the groups were balanced on the included covariates (Figure S2). After weighting, 51% of the amyloid PET group with CSF measures were amyloid positive, and 48% in the no‐PET group were.

**TABLE 1:** Baseline characteristics prior to weighting.

Characteristics	Total (*n* = 900)	Amyloid PET (*n* = 440)	No PET (*n* = 460)
Age at baseline in years, mean (SD)	63 (8)	64 (8)	62 (9)
Female, *n* (%)	392 (44)	174 (40)	218 (47)
Years of education,^a^ mean (SD)	12 (3)	12 (3)	11 (3)
MMSE, mean (SD)	24 (5)	25 (4)	24 (5)
CCI, mean (SD)	3 (2)	3 (1)	3 (2)
CSF available, *n* (%)	635 (71)	295 (67)	340 (74)
Syndrome diagnosis, *n* (%)			
SCD	364 (40)	164 (37)	200 (43)
MCI	135 (15)	83 (19)	52 (11)
Dementia	401 (45)	193 (44)	208 (5)

Abbreviations: CCI, Charlson Comorbidity Index; CSF, cerebrospinal fluid; MCI, mild cognitive impairment; MMSE, Mini‐Mental State Examination; PET, positron emission tomography; SCD, subjective cognitive decline; SD, standard deviation.

^a^ Years of education were calculated from the Verhage score.^31^

**FIGURE 2:**
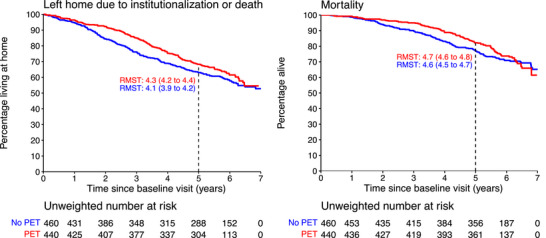
Survival curves and RMSTs for outcomes time living in community and time alive. RMST, restricted mean survival time (in years).

The restricted mean survival time over the first 5 years of follow‐up was larger for both outcomes in the PET group (Figure 2). Compared to the no‐PET group, PET group patients remained in the community on average 0.26 years longer (95% confidence interval [CI]: 0.05 to 0.46; approximately 3 months) and overall lived 0.15 years longer (CI: 0.02 to 0.27, nearly 2 months). The most pronounced deviation in the rate of leaving the community and mortality occurred in the first 3 years after diagnosis.

There was no significant difference in health insurance costs between the PET and no‐PET groups over 5 years of follow‐up (Table 2), at €703 (CI: −3974 to 5045) higher costs in the PET group. When also including institutionalization costs, total costs in the PET group were non‐significantly lower than in the no‐PET group (−€8258, CI: −20,622 to 3377). The largest mean difference in costs between the two groups, while not significant, was seen in institutionalization costs at −€8961 (CI: −20,438 to 2608) lower costs in the PET group due to less time spent institutionalized during the follow‐up period. Only in the mental health care cost category did the PET group have significantly lower costs (−€2144; CI: −4613 to −69).

**TABLE 2:** Disaggregated costs over 5 years.

Cost category	PET (95% CI)	No‐PET (95% CI)	Difference (95% CI)
**Health insurance costs**	30,533 (27,329 to 34,001)	29,829 (26,534 to 33,414)	703 (−3974 to 5045)
Medication	2601 (2176 to 3097)	3070 (2541 to 3666)	−469 (−1209 to 245)
Homecare	4948 (3565 to 6556)	5015 (3563 to 6745)	−67 (−2349 to 2151)
Hospital‐related care	12,794 (11,089 to 14,737)	11,873 (10,287 to 13,712)	921 (−1602 to 3402)
Primary care	2404 (2131 to 2707)	2538 (2302 to 2790)	−134 (−521 to 233)
Mental health care	3367 (2469 to 4386)	5511 (3806 to 7633)	−2144 (−4613 to −69)
Aid products	1046 (817 to 1328)	915 (705 to 1161)	130 (−209 to 478)
Other healthcare	872 (684 to 1095)	906 (700 to 1152)	−33 (−324 to 263)
Amyloid PET cost	2500	–	2500
Institutionalization	35,407 (27,908 to 43,542)	44,367 (36,199 to 53,330)	−8961 (−20,438 to 2608)
**Total costs**	65,939 (57,731 to 74,719)	74,197 (65,199 to 83,643)	−8258 (−20,622 to 3377)

*Note*: Primary care includes general practitioner costs, hospital‐related care includes in‐patient, out‐patient hospital, and geriatric rehabilitation costs (see Supplement 1 for full description and Dutch labels).

**FIGURE 3:**
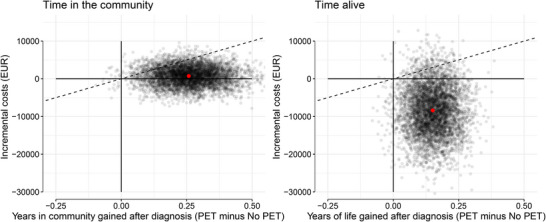
Cost‐effectiveness planes for time in community (left) and time alive (right) outcome. These figures display distribution of bootstrapped cost–effect pairs across the four quadrants of the cost‐effectiveness plane. Red dot: mean value for both years gained and incremental costs.

The point estimate of the ICER is 2708 per year gained living in the community, meaning that on average €2708 additional costs are incurred over 5 years to gain 1 year living in the community due to delayed institutionalization or death (Table 3). The cost‐effectiveness plane for time in the community (Figure 3) shows that 61% of the bootstrapped cost–effect pairs fall into the northeast quadrant, where the intervention is more effective but also more costly. Thirty‐nine percent of the cost–effect pairs are located in the southeast quadrant, suggesting that the intervention is more effective and less expensive. The cost‐effectiveness acceptability curves show the probability of cost‐effectiveness for different values of the WTP thresholds (Figure S3). The probability of adding amyloid PET being cost‐effective to gain 3 months in community is 39% at a WTP of €0 and increases to 76% at the elicited WTP of €2530.

**TABLE 3:** Results of cost‐effectiveness analyses.

Outcome	Perspective	RMST^a^ (years, 95% CI)	Cost difference^a^ (€, 95% CI)	ICER	NW (%)	SW (%)	NE (%)	SE (%)
Time in community	Health insurance	0.26 (0.05 to 0.45)	703 (−3974 to 5045)	2708	0	0	61	39
Time alive	Health insurance and institutionalization	0.15 (0.02 to 0.27)	−8258 (−20,622 to 3377)	−55,123	0	1	9	90

Abbreviations: CI, confidence interval; NE, northeast quadrant; NW, northwest quadrant; RMST, restricted mean survival time; SE, southeast quadrant; SW, southwest quadrant.

^a^ PET minus no PET.

For the outcome time alive, the ICER per year gained alive is −55,123, meaning that on average adding a PET scan to the diagnostic workup is dominant (cost‐saving and more effective) in the memory clinic population (Table 3). For this outcome, 90% of cost–effect pairs fall in the southeast quadrant, suggesting that adding PET to the diagnostic work‐up is associated with both longer survival and cost savings (Figure 3). Correspondingly, the probability of adding amyloid PET being cost‐effective to gain a year alive is 90% at a WTP of €0 (Figure S3). The sensitivity analysis based on propensity score matching is in line with the results presented in this section (Table S1 and Figure S4).

## 4 DISCUSSION

In this economic evaluation using data of a cohort of memory clinic participants with 5‐year follow‐up, we found that more accurate diagnosis resulting from the addition of amyloid PET to the diagnostic work‐up was associated with gains in time living in the community and longer survival. The gain in time living in the community in the PET group was not associated with a change in health insurance costs, but the longer survival time was accompanied by a trend toward lower total costs, largely attributable to a trend toward lower institutionalization costs. The uncertainty analysis suggested that there was a high probability that adding PET to the diagnostic work‐up was cost‐effective at the inferred WTP threshold for the outcome time living in the community and was dominant (i.e., more effective and less expensive) for the outcome time alive.

This study extends our previous work where we showed that adding an amyloid PET to the diagnostic work‐up in a memory clinic was associated with later institutionalization, lower mortality, and lower annual healthcare costs over a period of 4 years following diagnosis.^16^ These prior findings may be explained by studies showing amyloid PET enables a more accurate etiological diagnosis of AD^11^, ^12^, ^13^, ^14^, ^15^ and indications that appropriate disease management following a diagnosis could prevent subsequent health crises.^7^, ^9^, ^37^ The current study adds to our previous study by presenting a cost‐effectiveness analysis that shows that adding an amyloid PET to the diagnostic work‐up could be cost‐effective. However, these results must be interpreted with caution considering the non‐randomized design of our study as the participants could choose to participate in the diagnostic amyloid PET study. To adjust for imbalances between the groups, we performed inverse probability weighting. The sensitivity analysis using a different adjustment technique of propensity score matching found similar results, indicating successful adjustment for measured confounding. Nonetheless, we cannot exclude the possibility that differences between the PET and no‐PET group were caused by unmeasured confounding such as participants with higher health literacy choosing to undergo the PET or differences in baseline disease severity not accounted for by MMSE and functional independence (SCD, MCI, or dementia). We only included adjustment for education in our analysis, which was unlikely to fully capture health literacy. As better health literacy is associated with better health outcomes, this could be a driver of our results.^38^ Without randomization of PET receipt, negating both measured and unmeasured confounding, the cause of our findings remains uncertain.

Previous studies that assessed the cost‐effectiveness of more precise diagnosis of dementia were conducted using health economic models.^18^, ^19^, ^20^, ^21^ This study is the first empirical study to evaluate the long‐term cost‐effectiveness of adding amyloid PET to a diagnostic work‐up. It is based on high‐quality clinical data linked to the national registries to enable uniquely long follow‐up and avoid missingness in the outcome data. With this follow‐up length we add to the findings of the large IDEAS study. They analyzed Medicare data and found no significant differences in hospitalizations, emergency room visits, or total healthcare costs based on PET receipt 1 year after diagnosis.^17^ Our longer follow‐up revealed that the most pronounced impact on institutionalization and mortality occurred in the first 3 years after diagnosis, indicating IDEAS was potentially too brief to find an effect of amyloid PET on these endpoints.

There are limitations to consider in this study, in addition to its observational design. First, amyloid PET was provided in addition to the standard diagnostic work‐up, which also included lumbar puncture in a substantial number of patients, potentially reducing the added diagnostic benefits of the PET compared to situations where CSF analysis is not performed. Consequently, this would lead to an underestimation of the association between amyloid PET and time in the community, time alive, and costs in a setting where a PET would be the only indicator of AD pathology. Second, a young tertiary memory clinic cohort was used in this study. While this is representative of patients with high diagnostic uncertainty who are likely to benefit from adding an amyloid PET to the diagnostic work‐up, it limits the representativeness to a general incident dementia population. Third, the cost analyses were performed from a healthcare perspective and did not incorporate informal care costs or home care assistance and day care covered under the long‐term care act (in Dutch: Wet Langdurige Zorg). As these services likely partially substitute for the care provided in institutions, the cost difference between the groups from a societal perspective would probably diminish. Fourth, institutionalization costs in the amyloid PET group might have occurred outside of the 5‐year window of the cost‐effectiveness analysis due to a delay in institutionalization, overstating the reduction in costs due to amyloid PET over a lifetime. However, the difference between the restricted mean survival time of time alive and time in the community is lower in the PET group than in the no‐PET group, indicating the PET group spends less time institutionalized on average.

In conclusion, the results of this study suggest that a more accurate diagnosis by adding amyloid PET to the diagnostic work‐up may be cost‐effective in a cohort of patients referred to a tertiary memory clinic because of cognitive complaints. These observational results should be confirmed in randomized studies with long follow‐up to add strong causal evidence. Future empirical and modeling studies investigating the cost‐effectiveness of PET scans accounting for the emergence of disease‐modifying treatments are warranted.

## CONFLICT OF INTEREST STATEMENT

P.J. van der Veer, H.M. Broulikova, J.H. Hoogland, A.C. van Harten, J.E. Bosmans, and J. Berkhof declare no conflicts of interest. I.S. van Maurik received funding form ZonMW and STI‐MAG and a consulting fee from Roche (paid to the institution)

E. van de Giessen has performed contract research for Heuron Inc., AC Immune, and Roche. EvdG has a consultancy agreement with IXICO and Life Molecular Imaging for reading PET scans. All support was paid to the institution. W.F. has been an invited speaker at Biogen MA Inc., Danone, Eisai, WebMD Neurology (Medscape), NovoNordisk, Springer Healthcare, European Brain Council. W.F. is consultant to Oxford Health Policy Forum CIC, Roche, Biogen MA Inc., and Eisai. W.F. served on advisory boards of Biogen MA Inc., Roche, and Eli Lilly. W.F. is a member of the steering committee of phase 3 EVOKE/EVOKE+ studies (NovoNordisk). All funding is paid to her institution. W.F. is a member of the steering committee of PAVE and Think Brain Health. W.F. is a member of the Scientific Leadership Group of InRAD. W.F. was an associate editor of Alzheimer, Research & Therapy in 2020/2021. W.F. is an associate editor at Brain and a member of the Supervisory Board (Raad van Toezicht) of Trimbos Instituut.

## CONSENT STATEMENT

All participants provided written informed consent for their data to be used for research purposes.

## Supporting information



Supporting Information

Supporting Information
