# Supplementary material for: Long‐term cost‐effectiveness of a more accurate diagnostic work‐up for dementia
Source: Alzheimers Dement (Amst). 2025 Nov 4;17(4):e70210. doi: 10.1002/dad2.70210 (PMC12583976; doi:10.1002/dad2.70210)
Supplement: Supplementary file 1 — Supporting Information [file DAD2-17-e70210-s002.docx]

**Supplemental Methods**

In this study, cost information gathered by the Dutch Central Bureau of Statistics (CBS) was used. Medical costs reimbursed through universal health care is grouped by the CBS into several categories, which we further condensed. The condensed groups are outlined below.

*Primary care*

Primary care is a combination of the general practitioner (‘huisarts’), dental (‘mondzorg’), paramedic (‘paramedisch’), and multidisciplinary (‘multidisciplinair’). General practitioner (GP) costs are all costs made by the GP. Includes the standard reimbursements GPs receive per patients, costs per consult, and support in primary care. Dental costs are all reimbursed costs through universal health care in the Netherlands. Included in this is dental care for children below 18 and dental prosthesis. For example, filling a tooth is not reimbursed for adults under universal health care, so is not included in this category. Paramedic costs are all reimbursed paramedic care under the universal health care. This includes physiotherapy, speech therapy, occupational therapy and a dietetics. Multidisciplinary care costs includes care for patients with chronic disease (type 2 diabetes, vascular risk management, chronic obstructive pulmonary disease, asthma) in which multiple disciplines provide coordinated care.

*Hospital-related care*

Intramural is a combination of hospital care (‘ziekenhuis’) and geriatric care (‘geriatrisch’). Hospital care costs are all costs for specialist care in a hospital or independent specialist practice. Costs are counted for the year in which a treatment traject (‘diagnose-behandelcombinatie) is initiated, even if it extends into the next year. Geriatric care costs are cost relating to geriatric rehabilitation.

*Medication*

Medication only contains the medication (‘farmacie’) category. This category contains all reimbursed medication costs. This means some supplements are not included in these costs.

*Homecare*

Homecare only contains the homecare (‘wijkverpleging’) category. In this category there is costs relating to home care and nursing

*Aid products*

Aid is a combination of aid products (‘hulpmiddelen’) and sensory disability care (‘zintuigelijk’). Aid products are therapeutic aid products including things like crutches and hearing aids. Sensory disability care costs are reimbursements for extramural multidisciplinary care for people with a visual or auditory impairment, or communication deficit due to a developmental language disorder.

*Mental health care*

Mental health care is a combination of basic mental health care (‘basic GGZ’), and specialist mental health care (‘specialistische GGZ’). Basic mental health care costs are costs for light to moderate and or non-complex mental health care. Specialist mental health care costs are costs for severe or complex mental health care. This includes stay in intra- and extramural care. Costs are counted for the year in which a treatment traject (‘diagnose-behandelcombinatie) is initiated, even if it extends into the next year.

*Other*

Other is a combination of other (‘overig’), transport (‘ziekenvervoer’), care in foreign countries (‘buitenland’), and primary care stay (‘eerstelijnsverblijf’). Other is a category of costs not encompassed in a different category within Statistics Netherlands. Until 2015, multidisciplinary care was in this category. In 2017 primary care stay was in this category (the first year it was reimbursed under universal care). From 2018 onward primary care stay has its own category. Transport are costs related to transport in a situation of medical need ranging from acute transport for ambulance to accompanied transport for people with reduced vision. Foreign country care is care taking place in a different country. Primary care stay costs are costs for short term stay in a care facility. This also includes palliative care.

Statistical methods

To construct the inverse probability weights a logistic regression model was used with the amyloid PET receival as outcome and age, sex, MMSE, syndrome diagnosis, level of education, availability of cerebrospinal fluid biomarkers, and Charlson Comorbidity Index as covariates. With the logistic regression model, the propensity score (PS) of ‘exposure’ to an amyloid PET was calculated. Propensity scores were recalculated after excluding individuals with scores above 0.85 and below 0.15. For the amyloid PET group, the weights corresponded to 1/PS, and for the no-PET group, the weights corresponded to 1/(1-PS). No stabilization of weights was performed beyond trimming to achieve overlap in the PS.

**Supplemental results**


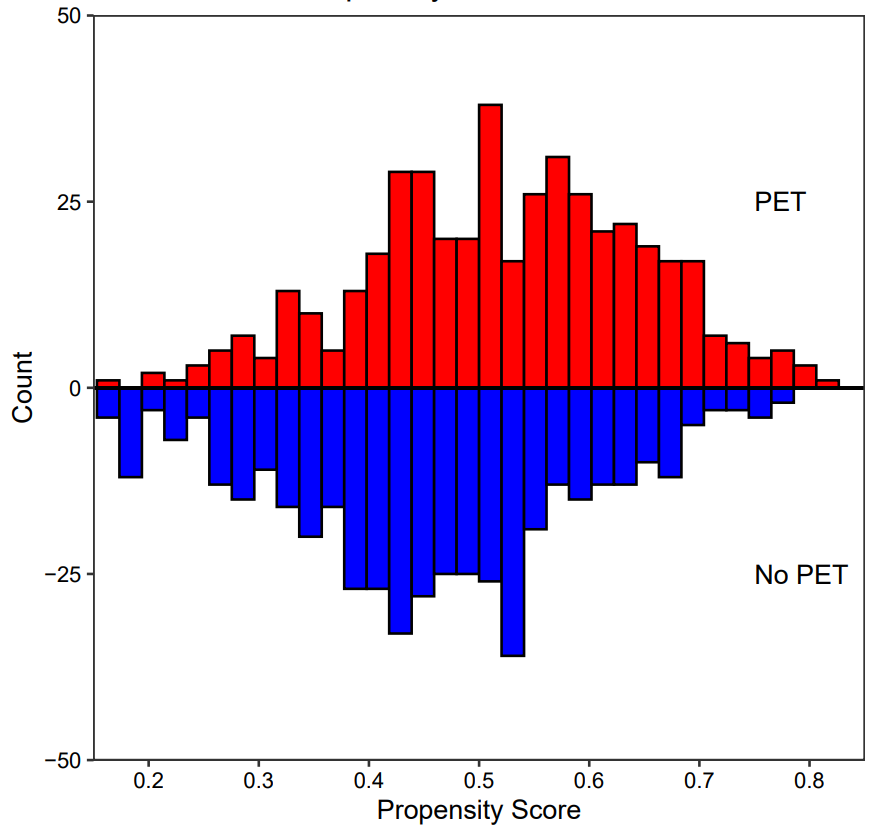


**Supplemental figure 1**: Distribution of propensity score in the amyloid PET and no-PET groups.

**Supplemental Figure 2**: Absolute standardized differences before and after inverse propensity weighting

Abbreviations: MMSE = Mini-Mental State Examination, CCI = Charleson Comorbidity Index, CSF = Cerebrospinal fluid (availability), SCD = Subjective cognitive decline, MCI = Mild cognitive impairment.


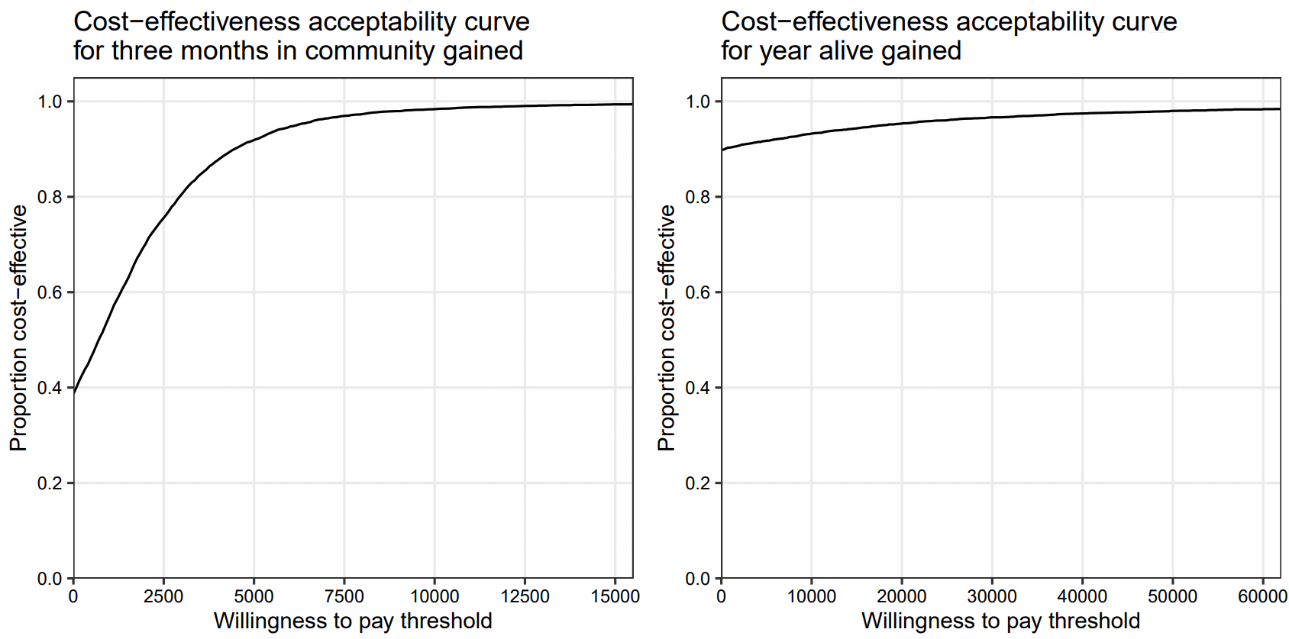


**Supplemental Figure 3:** Cost-effectiveness acceptability curve for the time in the community (left) and time alive (right) outcomes

Note: This figure displays the probability of the amyloid PET being cost effective for different willingness to pay thresholds. It is derived from the bootstrapped cost-effect pairs shown in Figure 4.

**Supplemental table 1:** Results of the cost-effectiveness analyses based on matched sample

| Outcome | Perspective | RMST (years, CI) | Cost difference (€, CI) | ICER | NW (%) | SW (%) | NE (%) | SE (%) |
| --- | --- | --- | --- | --- | --- | --- | --- | --- |
| Time in community | Healthcare | RMST: 0.30 (0.05 to 0.60) | -1161 (-8676 to 5146) | -4082 | 0 | 0 | 37 | 63 |
| Time alive | Healthcare and institutionalization | RMST: 0.27 (0.08 to 0.72) | -9850 (-25251 to 5223) | -33103 | 0 | 0 | 10 | 90 |

Note: RMST – restricted mean survival time; CI – confidence interval; NW – north-west quadrant of the cost-effectiveness plane; SW – south-west quadrant; NE – north-east quadrant; SE – south-east quadrant

**
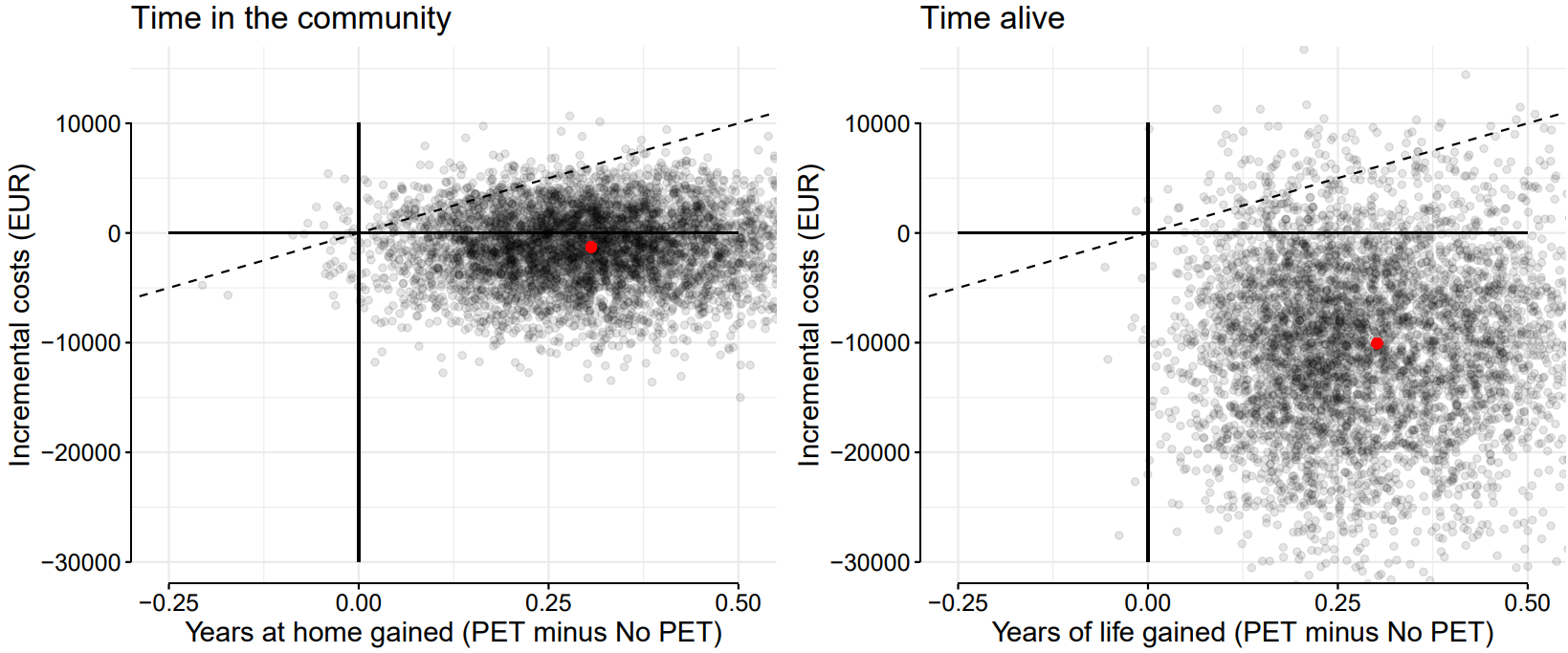
**

**Supplemental figure 4**: Cost-effectiveness planes for time in the community and time alive based on matched sample

Note: These figures display the distribution of bootstrapped cost-effect pairs across the four quadrants of the cost-effectiveness plane
